# Financial Incentives for Preventing Postpartum return to Smoking (FIPPS): study protocol for a three-arm randomised controlled trial

**DOI:** 10.1186/s13063-021-05480-6

**Published:** 2021-08-02

**Authors:** M. Ussher, C. Best, S. Lewis, J. McKell, T. Coleman, S. Cooper, S. Orton, L. Bauld

**Affiliations:** 1 Institute for Social Marketing and Health, University of Stirling, Stirling, FK9 4LA UK; 2 Population Health Research Institute, St George’s, University of London, SW17 ORE, London, UK; 3 Division of Epidemiology and Public Health, Medical School, University of Nottingham, Nottingham, NG7 2UH UK; 4 Division of Primary Care, Medical School, University of Nottingham, Nottingham, NG7 2UH UK; 5 Usher Institute, University of Edinburgh, Edinburgh, EH8 9AG UK

**Keywords:** Intervention, Randomised controlled trial, Pregnancy, Postpartum, Smoking relapse prevention, Smoking cessation, Financial incentives

## Abstract

**Background:**

Financial incentives are an effective way of helping women to stop smoking during pregnancy. Unfortunately, most women who stop smoking at this time return to smoking within 12 months of the infant’s birth. There is no evidence for interventions that are effective at preventing postpartum smoking relapse. Financial incentives provided after the birth may help women to sustain cessation. This randomised controlled trial will assess the effectiveness and cost-effectiveness of financial incentives to help women who are abstinent from smoking at end-of-pregnancy to avoid return to smoking up to 12 months postpartum.

**Methods:**

This is a UK-based, multi-centre, three-arm, superiority, parallel group, individually randomised controlled trial, with 1:1:1 allocation. It will compare the effectiveness of two financial incentive interventions with each other (one intervention for up to 3 months postpartum offering up to £120 of incentives (£60 for the participant and £60 for a significant other support); the other for up to 12 months postpartum with up to £300 of incentives (£240 for the participant and £60 for a significant other support) and with a no incentives/usual care control group. Eligible women will be between 34 weeks gestation and 2 weeks postpartum, abstinent from smoking for at least 4 weeks, have an expired carbon monoxide (CO) reading < 4 parts per million (ppm), aged at least 16 years, intend remaining abstinent from smoking after the birth and able to speak and read English.

The primary outcome is self-reported, lapse-free, smoking abstinence from the last quit attempt in pregnancy until 12 months postpartum, biochemically validated by expired CO and/or salivary cotinine or anabasine. Outcomes will be analysed by intention-to-treat and regression models used to compare the proportion of abstinent women between the two intervention groups and between each intervention group and the control group. An economic evaluation will assess the cost-effectiveness of offering incentives and a qualitative process evaluation will examine barriers and facilitators to trial retention, effectiveness and implementation.

**Discussion:**

This pragmatic randomised controlled trial will test whether offering financial incentives is effective and cost-effective for helping women to avoid smoking relapse during the 12 months after the birth of their baby.

**Trial registration:**

International Standard Randomised Controlled Trial Number 55218215. Registered retrospectively on 5th June 2019

**Supplementary Information:**

The online version contains supplementary material available at 10.1186/s13063-021-05480-6.

## Administrative information

Note: the numbers in curly brackets in this protocol refer to SPIRIT checklist item numbers. The order of the items has been modified to group similar items (see http://www.equator-network.org/reporting-guidelines/spirit-2727-statement-defining-standard-protocol-items-for-clinical-trials/).

**Table:** 

Title {1}	Financial Incentives for Preventing Postpartum return to Smoking (FIPPS): study protocol for a three-arm randomised controlled trial
Trial registration {2a and 2b}.	International Standard Randomised Controlled Trial Number registry, No. 55218215 10.1186/ISRCTN55218215 Registered retrospectively on 5th June 2019,
Protocol version {3}	V7.0, 16th November 2020
Funding {4}	Greater Manchester Combined Authority
Author details {5a}	^1^Institute for Social Marketing and Health, University of Stirling, Stirling, FK9 4LA, UK. ^2^Population Health Research Institute, St George’s, University of London, London SW17 ORE, UK. ^3^Division of Epidemiology and Public Health, Medical School, University of Nottingham, Nottingham NG7 2UH, UK. ^4^Division of Primary Care, Medical School, University of Nottingham, Nottingham NG7 2UH, UK. ^5^Usher Institute, University of Edinburgh, Edinburgh EH8 9AG, UK.
Name and contact information for the trial sponsor {5b}	Rachel Beaton Research and Development University of Stirling, Stirling FK9 4LQ, UK Rachel.beaton@stir.ac.uk
Role of sponsor {5c}	The University of Stirling is the trial sponsor and has delegated all responsibility for the management of the trial and publication of the findings to the Chief Investigator and co-investigators. The sponsor played no part in study design; collection, management, analysis and interpretation of data; writing of this or other reports or the decision to submit reports for publication.

## Introduction

### Background and rationale {6a}

More women stop smoking during pregnancy than at any other time; around half of pregnant women who smoke are likely to cease smoking ‘spontaneously’ [1]. This presents a valuable opportunity to help women who stop smoking in pregnancy to stop permanently. Most women who stop smoking in pregnancy wish to remain abstinent after the birth [2]. However, up to three-quarters of spontaneous quitters return to smoking within 6 months of the birth [3], thereby increasing their risks of smoking-related illness, as well as their children’s risks of passive smoking [4, 5] and of becoming smokers [6]. Behavioural support has been evaluated as an intervention for preventing postpartum relapse to smoking but there is no evidence for these interventions [7]. During pregnancy, offering financial incentives has been shown to be one of the most effective smoking cessation interventions [1, 8] and it is plausible that offering incentives in postpartum will reduce relapse to smoking.

Several studies have recruited women in early pregnancy and examined the combined effect of offering incentives in pregnancy and postpartum on postpartum rates of smoking abstinence. First, a trial that randomised women to incentives or a control group during pregnancy (*N* = 43) showed that, when providing an incentive voucher of $50 at 1 month and 2 months postpartum (contingent upon validated abstinence), around a third of women still relapsed by 2 months postpartum and the rate of relapse was similar in the control group [9]. Secondly, a non-randomised study (*N* = 58) offered a $20 incentive voucher (contingent upon abstinence) once weekly during the initial 4 weeks postpartum and then every other week for a further 8 weeks [10]. In the incentives vs control group, rates of abstinence reduced between end-of-pregnancy (37% vs 9%) and 24-week postpartum assessment (27% vs 0%), in favour of the incentives group. A further trial, which randomised women to receive incentives or not in pregnancy (*N* = 77), used the same intervention schedule as Higgins et al. [10] except that payments could escalate based on maintaining abstinence [11]. This study reported abstinence rates for incentives vs control at end-of-pregnancy of 41% vs 10% and at 24-week postpartum as 8% vs 3%. Higgins et al. (*N* = 118) [12] randomised women in pregnancy and included the same intervention as Heil et al. [11] as well as a revised condition which provided bonuses for those who could meet a more stringent biochemical validation criterion. The validated abstinence rates at end-of-pregnancy and 24 weeks postpartum, respectively, were standard incentives 36% vs 15%, revised incentives 45% vs 18% and control 18% vs 8%. In the largest study to date (*N* = 1014), women who were randomised in early pregnancy were offered cash incentives of $25–40 for each of four home visits at 1, 2, 4 and 6 weeks postpartum, $20 for each of five post-birth counselling calls and $40 for biochemically verified abstinence at 1 and 6 months postpartum [13]. At 6 months postpartum, 15% were validated as abstinent in the incentives group and 9% in the control group. This study did not report abstinence at end-of-pregnancy; therefore, it was not possible to examine the relapse rates. Finally, a pilot study (*N* = 60), randomising women in early pregnancy, used an app-based intervention and offered vouchers worth $33 for each validated assessment conducted twice weekly during the first 4 weeks postpartum and once weekly during a further 8 weeks postpartum [14]. At end-of-pregnancy, validated abstinence for the incentives vs control group was 37% vs 13%, while at 24 weeks postpartum it was 20% vs 7%.

While these studies show some potential benefit of postpartum incentives, randomisation was early in pregnancy for women who were currently smoking; therefore, it was not possible to examine the separate effects of postpartum incentives on smoking cessation. Moreover, all the studies were conducted in the US and all, except the study by Baker et al. [13], were limited by small sample sizes. We are conducting a UK-based large randomised controlled trial (RCT), which is the first to examine the specific effect of postpartum financial incentives on rates of postpartum relapse to smoking, among women who received incentives in pregnancy, were confirmed as abstinent at end-of-pregnancy and at that time were randomised to receive incentives or no incentives.

### Objectives {7}

#### Primary objective

The primary objective of this three-arm RCT is to assess the effectiveness of offering financial incentives to help women who are abstinent from smoking at end-of-pregnancy to avoid return to smoking up to 12 months postpartum.

#### Hypotheses

There are four hypotheses relating to the primary objective:

(i) That an intervention offering 1 months of postpartum financial incentives will be significantly more effective for aiding smoking cessation up to 12 months postpartum than a no incentives condition.
(ii) That an intervention offering 3 months of postpartum incentives will be significantly more effective for aiding smoking cessation up to 12 months postpartum than a no incentives condition.
(iii) That an intervention offering 12 months of postpartum incentives will be significantly more effective for aiding smoking cessation up to 12 months postpartum than an intervention offering 3 months of postpartum incentives.
(iv) That there will be a significant linear trend in smoking cessation across the three study groups, with rates of abstinence increasing from the no incentives group, to the 3 months incentives group, to the 12-month incentives group.

#### Secondary objectives

The secondary objectives are as follows:

- To assess the effectiveness of offering financial incentives to help women maintain smoking abstinence until at least 3 months after birth of the infant
- To assess whether financial incentives are cost-effective in terms of incremental cost per quitter at 12 months postpartum
- To identify the effects of maternal characteristics (e.g. cigarette consumption before pregnancy) and type of smoking cessation services (i.e. midwife-led or not) on the effectiveness of the intervention
- To explore participant’s and intervention provider’s experiences of and views on, the intervention and research procedures, in order to identify the barriers and facilitators to trial/intervention retention, effectiveness and implementation

### Trial design {8}

This study is a multi-centre, three-arm, superiority, parallel group, individually randomised controlled trial, with 1:1:1 allocation, designed to test the effectiveness of offering financial incentives to help women, who are abstinent from smoking at end-of-pregnancy, to avoid returning to smoking up to 12 months postpartum. In addition, an economic evaluation will assess the cost-effectiveness of offering financial incentives, and a qualitative process evaluation will examine barriers and facilitators to trial retention, effectiveness and implementation.

## Methods: participants, interventions and outcomes

### Study setting {9}

Study participants will be recruited from stop smoking services (SSS) serving maternity hospitals in four UK National Health Service (NHS) hospital Trusts in Greater Manchester. All participating Trusts cover large areas of deprivation and include a city, several provincial towns, suburban and rural areas. This diversity facilitates recruitment of women from different socio-economic backgrounds. Each of these sites have different SSS configurations offering their own care pathway within the framework of the UK National Institute for Health and Care Excellence (NICE) guidance [15]. This includes NHS/local authority-run services, generic/specialist pregnancy services and midwifery/SSS advisor-led services and represents most UK usual care pathways for smoking cessation in pregnancy. This also includes multiple care settings, from low-risk midwifery led care to high-risk tertiary units. The total deliveries per year at the four Trusts in 2019–2020 was 28,270, ranging from 2230 births in the smallest Trust to 12,150 in the largest Trust. In these Trusts, again in 2019–2020, 10.7% of women were recorded as smokers at time of delivery, against an average across England of 10.4%.

### Eligibility criteria {10}

#### Site eligibility: current routine care for smoking cessation in pregnancy

Study sites are only eligible if they are currently offering the Smokefree Pregnancy Programme in Greater Manchester, which incorporates an offer of financial incentives for biochemically validated smoking abstinence through to the end of pregnancy. This section describes this routine smoking cessation care which is offered in Greater Manchester.

This programme includes ‘BabyClear’ [16], which implements UK guidance [15] around smoking in pregnancy, as well as linking NHS Trusts who provide antenatal care and community-based providers of smoking cessation support. Training is provided for maternity staff, smoking cessation advisors and administrators within smoking cessation services. Midwives are trained to use CO monitoring for all women at the first antenatal appointment, with routine opt-out referral for smoking cessation support for any woman with a CO recording above 3 (parts per million) ppm. Babyclear also includes an enhanced ‘risk perception’ intervention for women who continue to smoke at their first trimester ultrasound scan appointment, at around 12 weeks gestation.

Women who report currently smoking, or having stopped smoking in the last 2 weeks, and have an expired CO reading > 3 ppm are offered regular (at least 4 weekly) support from a stop smoking advisor. Women can be referred by maternity services to receive this support or can self-refer. Expired CO is routinely tested at all smoking cessation appointments and stopping smoking is facilitated at the earliest possible point in pregnancy, with routes back into support at any point of relapse.

In addition to Babyclear, all the women in the pregnancy programme are offered a financial incentives scheme. This scheme is routine care and is not part of the intervention for the trial. For the first 4 weeks women are offered a £10 ‘Love2Shop’ voucher (redeemable in many UK retail outlets) per week and then a £20 voucher each month, up to 36 weeks gestation. This is conditional on the woman reporting lapse-free abstinence since their quit date and having an expired CO reading of < 4 ppm. Additionally, participating women are given the option to recruit a ‘Significant Other Supporter’ (SOS) (a member of their community, including a close family member such as a partner, who agrees to support the woman to remain smoke free, including attending cessation support sessions) who is entitled to receive a £60 Love2Shop voucher if the woman remains quit at 36 weeks gestation and the SOS is also confirmed as abstinent (CO < 8 ppm). If a woman relapses to smoking and agrees to set a new quit, she can be re-recruited into the incentives scheme, up to 32 weeks gestation. If she relapses a second time she is no longer eligible to participate in the scheme and is offered support outside the scheme. The latest point at which a women can begin a quit attempt as part of the Smokefree Pregnancy Programme/Babyclear is at 32 weeks gestation. If a woman is referred after 32 weeks she is offered routine support for smoking cessation in pregnancy without the offer of incentives (and is not eligible for the trial). In cases of miscarriage, premature birth or stillbirth the woman is no longer eligible for the incentives scheme and is offered cessation support outside of the scheme.

All women provided with smoking cessation support in pregnancy are also given brief advice about maintaining abstinence postpartum and in the long-term.

### Participant eligibility

Women who have undergone the above Smokefree Pregnancy Programme in Greater Manchester will be eligible to join the study if they meet the following inclusion criteria: are between 34 weeks gestation and 2 weeks postpartum, confirm having not smoked a single puff of a cigarette for at least 4 weeks, expired carbon monoxide (CO) reading < 4 ppm (piCO^baby^™ Smokerlyzer®, Bedfont Scientific Ltd.), aged at least 16 years, intend remaining abstinent from smoking after the birth (or to continue remaining abstinent if recruited postpartum), able to speak and read English and be willing and able to give written informed consent for participation in the study. If the participant is required to use a single-person, self-administered carbon monoxide monitor (iCO) (i.e. during COVID-19 restrictions), they require a device (e.g. phone) that is compatible with the monitor app.

#### Rationale for CO cut-off of < 4 ppm

During pregnancy, when metabolism is higher and there are respiratory changes, a CO cut-off of < 4 ppm is recommended [17, 18], whereas out of pregnancy a cut-off of < 8 ppm is more standard [19]. Therefore, for eligibility, we used a criterion of < 4 ppm. It is not clear whether metabolism and respiratory function would have returned to non-pregnant levels during the first 2 weeks postpartum compared with pregnancy; therefore we will apply the CO cut-off of < 4 ppm equally to women who are recruited in late pregnancy and to women who are recruited in the first 2 weeks postpartum.

There is recent evidence that the iCO can produce a slightly higher CO reading than more conventional CO monitors [20]. However, the iCO has not been compared with other monitors during pregnancy; therefore, we retained the cut-off of < 4 ppm for the iCO during testing for eligibility.

### Who will take informed consent? {26a}

All women confirmed as abstinent by their stop smoking service (SSS) advisor at around 36 weeks gestation (acceptable range, 34 weeks gestation to 2 weeks postpartum), and meeting other eligibility criteria, will be invited by their advisor to join the study. The SSS advisor will explain that we are running an RCT examining the effects of offering shopping vouchers on women’s smoking cessation during the 12 months after their baby is born. If a face-to-face appointment is possible at this time, the SSS will ask women for their written informed consent to participate in the trial. They will also ask for their consent to be contacted after the 12 months follow-up to be invited to take part in an interview to discuss their experience of the trial. It will be made clear that women can participate in the trial without consenting to be interviewed and that this will not affect their participation in the trial or the usual care that they receive.

All women who are eligible and have provided consent to participate in the trial will be randomised to one of the three study groups. After women are randomised, they will be given an additional participant information sheet (PIS) describing the specific study group they have been allocated to.

All SSS staff will attend a half-day session, delivered by a member of the research team, to be informed about the trial and to be trained in taking consent, delivering the intervention and other research procedures.

#### ‘Distanced’ consent methods

Due to COVID-19 restrictions on face-to-face contact, the following provisions have been made: Potential participants can be contacted at around 32 weeks and 36 weeks gestation by phone, as well as face-to-face. The participant information sheet and consent form can be sent to participants by post, email or via a web-link embedded within a text message. Women will be offered several ‘distanced’ methods for providing informed consent:

1. To post the completed written consent form to a researcher or SSS before being randomised
2. To take a photo or scan of their completed written consent form and texting or emailing it to a researcher or SSS advisor
3. To provide verbal consent to join the trial. During the call, potential participants will give explicit verbal consent to the statements on the consent form. An SSS advisor will then sign the participant’s consent form and check a ‘tick box’ which notes on the consent form that consent was taken verbally by telephone.

#### Justification for distanced consent methods

We believe it is ethical to use the ‘distanced’ consent methods described above and they have received ethical approval. If all participants are required to receive, sign and return signed consent forms, this will result in many women, who wish to join the trial, not returning signed confirmation. For example, women may be socially isolating and do not wish to leave their homes to post the consent form or they may not have the facility to transfer the completed form electronically. In this circumstance adequate recruitment and, hence, a robust evaluation would be difficult. Moreover, a ‘hard-to-reach’ group would lose the potential to benefit from participation in the trial.

### Additional consent provisions for collection and use of participant data and biological specimens {26b}

No additional consent provisions are required.

### Interventions

#### Explanation for the choice of comparators {6b}

Those in the ‘no incentives’ control group will receive postpartum care as usual, except, as for all participants, they will be offered a £20 voucher payment, at both 3 months and 12 months postpartum, as a gesture of thanks for completing a follow-up assessment. During pregnancy, all women receive routine care for smoking cessation in pregnancy, including the offer of incentives, as described in section ‘Eligibility criteria {10}’. This includes brief advice about maintaining abstinence postpartum and in the long-term. Other than this, currently there is no routine, postpartum relapse prevention support offered to women or partners who have quit smoking before or during pregnancy.

#### Intervention description {11a}

There are two intervention/incentive groups. The incentive payments are in the form of Love2Shop gift cards that can be redeemed in a wide variety of UK shops and that are used in the pregnancy incentives scheme that the women have participated in. Love2Shop vouchers have been used in a previous UK trial of incentives for smoking cessation in pregnancy [21].

*During COVID-19 restrictions on face-to-face contact*, participants will attend appointments remotely, via telephone, rather than face-to-face and smoking cessation status will be assessed remotely. Also, vouchers will be posted to participants. See section ‘Outcomes {12}’ for details of ‘Assessing smoking cessation outcomes during COVID-19 restrictions on face-to-face contact’.

#### Intervention group 1: incentives are offered up to 3 months postpartum

This intervention is provided in addition to the usual care as received by the ‘no incentives’ group.

##### Payments to participants

Participants are offered a total of £60 of incentive payments. An incentive of £20 is offered at each of the visits at 1, 2 and 3 months postpartum. Payments are conditional on self-report of not smoking a single puff of a cigarette since their last quit date in pregnancy and an expired CO reading of < 8 ppm. This intervention, including the amount of incentive, was chosen because it has been used previously in the UK, including in Greater Manchester, and has been shown to be acceptable to postpartum women and SSS advisors [22].

##### Payments to significant other supporter

Participants will also be given the option to identify a ‘Significant Other Supporter’ (SOS) (a member of their community, including a close family member such as a partner, who agrees to support the woman to remain smoke free, including attending smoking cessation validation visits). The women’s SOS will be offered an incentive of £60 if the woman achieves CO validated abstinence at 3 months postpartum and the SOS is also confirmed as abstinent (CO < 8 ppm), irrespective of whether the SOS has been a smoker. During the period of COVID-19 restrictions on face-to-face contact, the SOS will be confirmed as abstinent based on self-report alone. Previously, SOS payments have been shown to be acceptable to postpartum women and their partners in the UK [22] and elsewhere [9].

The total value of incentives offered to this group, including payments to the participant (£60) and the SOS (£60), is £120.

### Intervention group 2: incentives are offered up to 12 months postpartum

This intervention is provided in addition to the usual care as received by the ‘no incentives’ group.

In addition to all the incentives offered to group 1, participants in this group are offered £60 at each of the visits at 6, 9 and 12 months postpartum. The rationale for the amount of incentive offered in the 12-month condition was based on repeating the payment made to the participant at 3 months (i.e. £60) at 3-month intervals (i.e. at 6, 9 and 12 months) up to 12 months. Again, payments are dependent on expired CO confirmation of self-reported abstinence. No additional incentive is offered to the SOS beyond that offered at 3 months.

The total value of incentives offered to this group, including those offered to the participant (£240) and SOS (£60), is £300. This intervention was devised specifically for this study, in order to examine the potential benefit of continuing the offer of incentives, at 3-month intervals, up to the primary end-point at 12 months postpartum.

To our knowledge, neither of the incentive interventions has been previously tested in a research study. The level of incentives offered in the interventions are between the lowest level suggested by the general public that might be effective (£20 per month) and the highest acceptable level (£80 per month), for during pregnancy [23]. No such data on incentives is available for postpartum.

### Behavioural support for intervention groups

In both intervention groups, the SSS advisor is instructed to provide only behavioural support that is integral to the incentives intervention. In total, the interventions include the following four behaviour change techniques (BCTs), as defined by a standard taxonomy of BCTs [24], and the numbering is that used in the taxonomy:

10.1 *Material incentive (behavior):* Inform that a financial incentive will be offered if the participant and SOS reports not smoking and has a CO < 8 ppm

1.3 Goal setting (outcome): Set a goal of not smoking a single puff of a cigarette until the next assessment

2.6 Biofeedback: Inform the person of their expired CO reading

10.4 Social reward: SSS congratulates person if they remain abstinent from smoking

Adherence to the protocol for providing behavioural support will be been explored in the interviews with smoking cessation advisors.

### Payments for attending assessments

In addition, in order to maximise completion of follow-ups, all participants are offered a £20 voucher payment, for completing a follow-up assessment at both 3 months and 12 months postpartum (i.e. £20 for each assessment).

### Criteria for discontinuing or modifying allocated interventions {11b}

There are no special criteria for discontinuing or modifying allocated interventions.

Participants may choose to stop attending appointments and receiving incentive payments for any reason.

### Strategies to improve adherence to interventions {11c}

None beyond the brief behavioural support described in {11a}.

### Relevant concomitant care permitted or prohibited during the trial {11d}

Trial participants are permitted to receive relapse prevention support, during postpartum, beyond that provided by the intervention and any additional support is recorded. The SSS conducting the research follow-ups are asked not to provide any relapse prevention support at the follow-ups beyond that defined as part of the intervention (for the description of the allowed support see: {11} Intervention description/Behavioural support for intervention groups).

### Provisions for post-trial care {30}

None, beyond standard care within the NHS

### Outcomes {12}

At the 3 and 12 months postpartum follow-ups, women will initially be assessed for smoking status over the phone (up to five attempts will be made to call them) by their stop smoking service (SSS) advisor or by a researcher. Those who report having not smoked a single puff of a cigarette since their last quit date in pregnancy will be asked to attend a face-to-face appointment with their SSS advisor to confirm their smoking status or to confirm their smoking status by remote means (see section ‘Assessing smoking cessation outcomes during COVID-19 restrictions on face-to-face contact’).

### Primary outcome measure

The primary outcome is self-reported, lapse-free, smoking abstinence from the last quit attempt in pregnancy until 12 months postpartum, biochemically validated by an exhaled CO reading of < 8 ppm, and/or saliva cotinine or anabasine estimation. Where possible, both a CO reading and saliva sample will be collected and for a woman to be validated as abstinent both the CO reading and saliva test will need to confirm abstinence. Where both a CO reading and saliva sample cannot be collected, either a CO reading *or* a saliva test will be used to confirm abstinence. The proportion of abstinent women will be compared between the two intervention groups and between each intervention group and the control group. During pregnancy, due to physiological changes including higher metabolism [25], a CO cut-off of < 4 ppm is recommended [17, 18], whereas out of pregnancy a cut-off of < 8 ppm is more standard [19].

Where the expired CO reading is < 8 ppm, or a CO reading is not available, and the cotinine level is < 10 ng/ml [26], the participant is defined as a biochemically verified as abstinent. Where the CO reading is < 8 ppm, or a CO reading is not available, and the cotinine level is ≥ 10 ng/ml and the participant reports use of nicotine replacement therapy, e-cigarettes or heat-not-burn products, then saliva samples will be also tested for anabasine. Where the anabasine result is < 0.2 ng/ml [27], then the participant will be defined as a biochemically verified as abstinent.

Since recruitment to the study is conducted between 34 weeks gestation and 2 weeks postpartum, it is possible that a few women may relapse after recruitment and before giving birth. In the analysis for the primary outcome these women will not be distinguished from those who have relapsed after the birth. However, we will report the numbers of women who relapsed before the birth, separately for the two study groups.

### Secondary outcome measures

As a secondary outcome we will assess self-reported, lapse-free, smoking abstinence from the last quit attempt in pregnancy until 3 months postpartum, biochemically validated by an exhaled CO reading of < 8 ppm. Again, the proportion of abstinent women will be compared between the intervention and control groups. At both 3 and 12 months, for some women it may not be possible to conduct a test to biochemically validate self-reported abstinence (e.g. due to COVID restrictions). Therefore, as further secondary outcomes we will compare self-reported abstinence between the study groups at 3 and 12 months (including without biochemical validation as well as those with validation).

If a participant is recorded as having relapsed at 3 months postpartum, they will be automatically recorded as having relapsed for the primary outcome at 12 months postpartum, and will not be followed-up at 12 months.

In addition, in order to understand any differences between study groups, we will assess the following measures at three and 12 months postpartum (see Table 1):

**Table 1:** Schedule of assessments

Assessment	Baseline	3 months postpartum	12 months postpartum
Demographics: age, education, occupation, ethnicity	X		
Smoking status: self-reported	X	X	X
Time since last smoking	X		
Expired CO level in ppm	X	X	X
Use of iCO single-person use CO monitor outside of assessments: self-reported		X	X
Cigarette consumption before pregnancy	X		
Self-efficacy for smoking cessation	X		
Use of nicotine products	X	X	X
Smoking in the home	X	X	X
Partner smoking status	X	X	X
Use of additional smoking cessation support	X	X	X
Whether they have a significant other supporter (SOS)	X		
Gestation	X		
Parity	X		
Breastfeeding intent	X		
Depression (Edinburgh Postnatal Depression Scale)	X		
Alcohol consumption: Alcohol Use Disorders Identification-Consumption (AUDIT-C) test	X		
Expired CO level in ppm	X	X	X
Saliva for cotinine/anabasine test			X

1. Use of nicotine products:
  - Have you used any nicotine replacement therapy (NRT) in the last week? Yes/no
  - If yes, which types of NRT have you mainly used? (Tick all that apply)
  - Patch/lozenge/mouth spray/gum/microtabs/inhalator/nasal spray
  - Have you used an e-cigarette in the last week? Yes/no
  - Have you used a heat-not-burn product in the last week (e.g. iQOS)? Yes/no

The assessment of use of nicotine products at 12 months postpartum also enabled us to consider the appropriateness of using a test of saliva cotinine vs anabasine, to determine smoking status, at this time.

- Smoking in the home: Does anyone living in your home smoke tobacco? Yes/no
- Partner smoking status: If you have a partner, does your partner smoke tobacco? Yes/no/not applicable
- Use of additional smoking cessation support beyond that provided in the trial:

Are you currently receiving any professional help with stopping smoking other than the help you receive as part of this research study? Yes/no; if yes, which organisation is giving you this help?

- Use of the iCO (single-person use) monitor beyond that required for the research assessments:

Have you used your iCO carbon monoxide monitor at times other than when asked to do so by your stop smoking advisor? Yes/no; if yes, on how many days have you used the iCO in this way since your baby was born? (Tick one box only) On 1 or 2 days/on 3 to 5 days/on 6 to 10 days/on 11 to 20 days/on more than 20 days

### Assessing smoking cessation outcomes during COVID-19 restrictions on face-to-face contact

During COVID-19, for the 3- and 12-month postpartum outcomes for smoking cessation, if it is not possible to take an expired CO reading during a face-to-face visit, participants will take a CO reading using a self-administered/single-person use device (iCO^TM^ Smokerlyzer, Bedfont Scientific Ltd.) [28] and send the CO reading to the stop smoking advisor via an app. There is recent evidence that the iCO can produce a slightly higher CO reading than more conventional CO monitors [20]. However, the authors of this study recommend an optimal cut-off for the iCO of < 6 ppm; therefore, we decided to retain our more liberal cut-off of < 8 ppm.

If a participant joined the trial before the COVID-19 restrictions and therefore was not sent a self-administered CO monitor when they were recruited and a face-to-face CO reading is not possible, then a CO reading will not be collected at 3 or 12 months follow-up; at 3 months, abstinence will be self-reported alone and at 12 months self-reported abstinence will be validated, where possible, by a saliva test. The participant will be sent a salivette and instructions to provide a saliva sample and will be advised how to take a saliva sample during the follow-up call with the stop smoking advisor. The participant will then be asked to post the saliva sample to a laboratory for analysis (ABS Laboratories, Hertfordshire, UK, https://www.acmgloballab.com/about-us/our-locations/europe-london-uk). The follow-up questionnaires will be administered by the participant’s stop smoking advisor over the phone. When the COVID-19 restrictions have been lifted, we will give participants the option of continuing with the above approach or they can choose to have face-to-face appointments.

### Economic measures

We will examine the incremental cost per quitter for the financial incentive interventions versus the no incentives group. We will collect resource and cost data (i.e. financial incentives, stop smoking service delivery) as well as considering rates of smoking cessation.

### Process evaluation

The process evaluation will collect quantitative data on recruitment and follow-up rates. In addition, qualitative data will be collected via a focus group with SSS advisors/managers delivering the intervention and through interviews with trial participants. This qualitative work will identify barriers and facilitators to trial recruitment and adherence and explore the acceptability of study processes and procedures. Full details of the process evaluation are reported in Additional file 1.

### Baseline measures

In a baseline questionnaire, administered by the SSS advisor before randomisation, we will assess measures which have been shown to predict postpartum smoking relapse [29] and which we would want to ensure are similar for the three study groups, as potential confounders of any effects on smoking cessation (see Table 1). These measures are:

- Age
- Ethnicity
- Highest educational qualification
- Occupation
- Gestation
- Level of cigarette consumption before pregnancy
- Expired CO level in ppm
- Parity (i.e. number of previous pregnancies that have gone beyond 24 weeks)
- Assessment of any smoking in the home and of whether partner smokes
- Length of time since last smoking (months/weeks)
- Self-efficacy: rating of ‘How confident are you that you will continue not to smoke at least until your baby’s first birthday?’ (not at all confident, slightly confident, moderately confident, very confident, extremely confident) [30]
- Depression: Edinburgh Postnatal Depression Scale [31] (for the item **‘The thought of harming myself has occurred to me**’**, if women respond ‘Yes, quite often’ or ‘sometimes’, they will be referred to** their health visitor, family nurse partnership or GP)
- Breastfeeding intent [32]
- Alcohol consumption: Alcohol Use Disorders Identification-Consumption (AUDIT-C) test [33, 34]
- Use of support for smoking cessation beyond what is provided in the trial
- Whether they recruited a significant other supporter (SOS) during their pregnancy
- Use of nicotine replacement therapy (NRT) in the last week and main types of NRT used
- Use of heat-not-burn products in the last week
- Use of e-cigarettes in the last week

### Participant timeline {13}

**Fig. 1:**
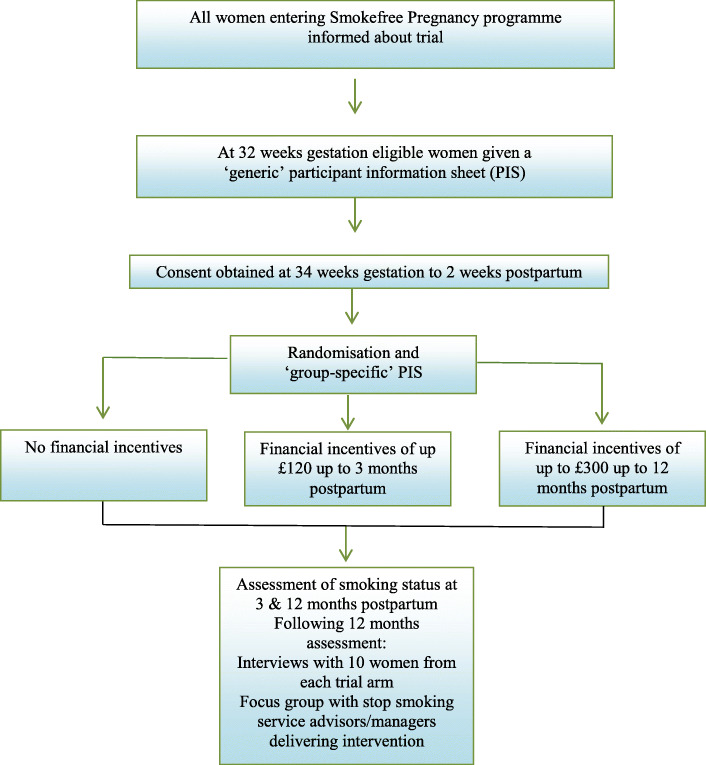
Trial flow diagram

The trial flow diagram is presented in Fig. 1.

#### Sample size {14}

We estimate that the proposed sample size of 900 women will give 90% power at an alpha threshold of 0.017 to detect a difference in abstinence rates across any two groups of 13.6% at 12-month follow-up. It is anticipated that the abstinence rate in the control group will be 9% at 12-month follow-up, that there will be 22.6% abstinence at 12 months follow-up in the 3-month incentives group and 36.2% abstinence at 12 months follow-up in the 12 months incentives group. The figure of 9% in the no incentives group is conservatively based on the assumption that over 90% of women without incentives may relapse by 12 months postpartum [3]. The estimate of 13.6% difference is based on the group difference found by Tappin and colleagues in a study of incentives during pregnancy [34]. The threshold for alpha is reduced to 0.017 to correct for multiplicity and allow comparisons across the three arms. This sample size will also give 100% power to detect a linear trend in abstinence across the three groups (*p* < 0.05) from control to 12-month intervention.

#### Recruitment {15}

At the time that women join the Smokefree Pregnancy programme, which is most commonly following their first antenatal booking visit at 8–12 weeks gestation, they will be informed by their stop smoking service (SSS) advisor that those who are confirmed as abstinent from smoking at around 36 weeks gestation (acceptable range, 34 weeks gestation to 2 weeks postpartum) will be offered the opportunity to join a research study in which they may or may not be offered shopping vouchers to remain abstinent through 12 months postpartum.

Women confirmed as abstinent (CO < 4 ppm) by their SSS at around 32 weeks gestation will be given or sent a ‘generic’ PIS about the study. We decided to first provide a generic PIS, explaining that incentives may or may not be offered, but not giving the specific details of the three study groups, in order to reduce potential dissatisfaction and dropout if women are not offered the highest value of incentives. It will be explained that if they are still confirmed as abstinent at their 36-week appointment, and meet other eligibility criteria, they will have the opportunity to discuss the study and decide if they want to join. All women confirmed as abstinent at around 36 weeks gestation by their SSS (acceptable range, 34 weeks gestation to 2 weeks postpartum), and meeting other eligibility criteria, will be invited by their SSS advisor to join the study. If women give birth before their 36 week appointment they will still be eligible as long they join the study within 2 weeks of the birth of the baby and meet other eligibility criteria. The SSS advisor will explain that we are running an RCT examining the effects of offering shopping vouchers on women’s smoking cessation during the 12 months after their baby is born and seek consent to participate. See {26a} for a description of the consent process.

A screening form will be completed by the SSS advisor to record number of women approached, eligibility, number of women declining to take part or not eligible, and reasons for being ineligible or declining, if women are willing to provide this information.

If a face-to-face visit and/or multiple use CO test is not possible (e.g. in order to maintain social distancing) the CO reading to determine eligibility will be taken by a single-person, self-administered CO monitor, posted to the woman following her appointment at 32 week’s gestation, and used by the woman at home (iCO^TM^ Smokerlyzer, Bedfont Scientific Ltd.) [28]. The CO reading will be transferred to the SSS advisor using an app. Women who do not have a device (e.g. phone) that is compatible with this app will not be eligible to join the trial. The baseline questionnaire will be administered by the stop smoking advisor over the phone. When the COVID-19 restrictions have been lifted, we will give participants the option of continuing with the above approach or they can choose to have face-to-face appointments.

Details of those consenting to participate in the trial will be entered by the SSS advisor on to a secure University of Stirling online server which will generate a randomisation code. Some participants may be disappointed not to be allocated to one of the incentives groups and the SSS advisors will be trained to respond appropriately to this. Following randomisation, the participant’s screening form, baseline questionnaire and consent form will be sent to the study principal investigator who will review this documentation and confirm eligibility.

The patient’s clinical care team (e.g. midwife, health visitor, GP) will already have been made aware that the woman is taking part in the Greater Manchester Smokefree Pregnancy programme; therefore, we will not be sending a separate letter to patient’s general physicians (GPs) when they join the trial.

### Assignment of interventions: allocation

#### Sequence generation {16a}

Randomisation and allocation will be carried out by the trial statistician (CB) at the University of Stirling using STATA, with randomised permuted blocks and stratified by site (i.e. hospital trust). Unit of randomisation will be individual participant, allocated in a ratio of 1:1:1 to the two intervention groups or control group. Following completion of the baseline questionnaire, details of women who consent to participate will be entered by the SSS advisor into a University of Stirling secure online server (i.e. randomisation date, participant birth date, participant initials and site). The SSS will then be automatically informed of the women’s unique participant number and treatment allocation and this information will be given to the participant. Selection bias will be minimised by ensuring all consenting women have equal opportunity of being allocated to each of the study arms.

#### Concealment mechanism {16b}

Randomisation will use a web-based randomisation system.

### Implementation {16c}

SSS advisors at each site will enrol participants and request the group allocation.

### Assignment of interventions: blinding

#### Who will be blinded {17a}

Due to the nature of the intervention, participants and the SSS advisors delivering the intervention will not be blinded to the treatment received. Nor will SSS advisors or researchers who ascertain smoking status at 3 and 12 months postpartum be blinded; however, there is low risk of bias as smoking status is biochemically validated. Those involved in the data analyses will be blinded to the group allocation.

#### Procedure for unblinding if needed {17b}

Participants and SSS staff delivering the intervention are not blinded.

### Data collection and management

#### Plans for assessment and collection of outcomes {18a}

Data will be collected via questionnaires at baseline and at three and 12 months postpartum. Questionnaires will be either self-completed by participants or, during COVID-19 restrictions, will be administered by SSS advisors over the telephone. Demographic information will be collected at baseline via questionnaire. CO readings will be collected at baseline and at 3 and 12 months postpartum during a face-to-face visit or, during COVID-19 restrictions, remotely via an app. For those who report smoking abstinence at 12 months postpartum saliva, for cotinine/anabasine testing, will be collected face-to-face or, during COVID-19 restrictions, saliva collection kits will be posted to participants and participants will post the saliva sample to the laboratory.

#### Plans to promote participant retention and complete follow-up {18b}

In order to maximise the number of participants who complete follow-ups, especially among those in the control group and those who have relapsed to smoking, all participants will be offered a £20 voucher payment, for completing a follow-up assessment at both 3 months and 12 months postpartum (i.e. £20 for each assessment). The assessment includes completion of a questionnaire and biochemical assessment of smoking status. A previous UK trial of incentives for smoking cessation in pregnancy showed that such a payment motivates women to attend follow-ups [29]. Telephone contact for follow-up will be attempted on up to five occasions. If a participant has been asked to post a saliva sample to the laboratory and the saliva sample has not arrived, the participant will be contacted and reminded to send the sample.

#### Data management {19}

All trial participants are given an individual trial number which will be used on all case report forms (CRF) for that participant. Researchers at the University of Stirling will enter data from CRFs into the secure trial database. To check for systematic errors, double data entry will be conducted for a random selection of 10% of CRFs. University of Stirling researchers will review CRFs and the database for range errors and for missing data.

#### Confidentiality {27}

All collected information will be kept strictly confidential and will be stored in accordance with the General Data Protection Regulation (GDPR) and the latest Directive on Good Clinical Practice (GCP). Confidentiality of patient’s personal data is ensured by not collecting patient names on CRFs and limiting access to personal information held on the databases. At trial enrolment, the participant will be issued a participant identification number and this will be the primary identifier for the participant, with secondary identifiers of month and year of birth and initials. Any paper copies of personal trial data will be kept at the participating site in a secure location with restricted access. Following consent, identifiable data will be kept in a secure database at each site and at the University of Stirling, to allow authorised members of the site team to contact participants in order to arrange appointments/assessments.

Any paper copies of consent form, with patient name and signature, will be kept securely at the trial site with a copy sent to the University of Stirling for monitoring purposes. Consent forms will not be kept with any additional patient data.

### Plans for collection, laboratory evaluation and storage of biological specimens for genetic or molecular analysis in this trial/future use {33}

None

### Statistical methods

#### Statistical methods for primary and secondary outcomes {20a}

The plan for statistical analysis is reported here in accordance with guidance for RCTS [35, 36].

We will use descriptive statistics to present the baseline characteristics of the three study groups. Descriptive statistics will also be used to report rates of recruitment, retention and follow-up at 3 months and 12 months postpartum and to report the numbers of women who relapsed before the birth vs after the birth, separately for the two study groups. Again, descriptive statistics will be used to present the following secondary outcomes at 3 and 12 months postpartum: use of nicotine products, smoking in the home, partner smoking status, use of additional smoking cessation support beyond that provided in the trial and use of the iCO (single-person use) monitor beyond that required for the research.

Analyses of smoking status, for primary and secondary outcomes, will be performed for the intention-to-treat population and reported in accordance with the Consolidated Standards of Reporting Trials (CONSORT) statement [37, 38], including all women who are randomised and meet the eligibility criteria. If ineligible persons are mistakenly randomised into the trial, the independent trial steering committee will review the case for removing the participant from the analysis, such that this decision is unbiased and not influenced by events that occurred after randomisation (and may therefore be affected by whether patients received experimental or control treatment) [39].

Women lost to follow-up will be presumed to have returned to smoking [19]. This assumption is standard in the smoking cessation literature as the pattern of missingness is not random. We will compare differences in smoking cessation outcomes between all three treatment groups, using logistic regression adjusted for the random effect of site (i.e. NHS Trust), with statistical significance determined by the likelihood-ratio test, and with pairwise comparisons between treatment groups in accordance with the study objectives, using a *p* value of 0.017 adjusted for the three comparisons. The estimate of effect will be given as the odds ratio and 95% confidence interval for each of the three comparisons. We will also look for a linear trend in smoking cessation across the three groups, by examining the significance (*p* < 0.05) of a linear contrast across the groups from control to 12-month intervention.

All outcomes will be analysed collectively following the completion of the final follow-up at 12 months postpartum. A more detailed statistical analysis plan will be agreed to before the end of data entry and before the treatment code is broken.

### Interim analyses {21b}

There will be no interim analyses.

### Methods for additional analyses (e.g. subgroup analyses) {20b}

We will conduct a secondary-analyses adjusting for key baseline maternal variables that are predicted to be related to smoking status (i.e. socio-economic status, cigarette consumption before pregnancy, depression and age) and type of smoking cessation service (midwife-led or not) [40]. We will also conduct sensitivity analysis to examine the effects of remote (i.e. during COVID-19) versus face-to-face consultations. We will also consider whether the women have been supported by a significant other.

### Economic analysis

An incremental cost-effectiveness analysis will be undertaken, following the NICE guidance for healthcare evaluations [41, 42], comparing the additional costs of the financial incentive interventions with those of the no incentives group, as well as the additional benefits, to give a cost per additional quitter. The economic evaluation will use resource and cost data (i.e. financial incentives, Stop Smoking Service delivery) as well as rates of smoking cessation.

We will document resources consumed that are related to each intervention, including the cost of personnel, staff training, materials, space, equipment and administrative overheads, as well as the costs of the financial incentives. Data collection methods will include (i) accounting for staff time using time and effort reports; (ii) accounting for computer time, mailing and program costs using an accounting system that has been created to facilitate real-time aggregation of these costs; and (iii) using information gathered in the focus group with staff to determine the amount of time they devote to tasks related to the incentives intervention. All resources identified during the study will be valued using appropriate local and national unit cost data. The values used will be the most up to date at time of analysis, if unit cost data are obtained from more than one year then appropriate inflators will be used to transform costs into a common cost year.

The analysis will adopt a ‘within trial’ approach (i.e. up to the 12 months follow-up point of the trial). The main outcome measure used in the economic analysis will be the study’s primary outcome measure, lapse-free, biochemically validated smoking abstinence at 12 months postpartum. This will allow us to examine incremental cost per additional quitter. Multiple imputation will account for missing data assuming data are missing at random, so that missingness can be predicted by other complete cases.

Costs and effects will be analysed using regression-based methods to allow for any differences in baseline characteristics. Incremental costs and effects will be reported. Additionally, if one group is more costly and more effective than the other we will report incremental cost-effectiveness ratios (ICERs). Non-parametric bootstrapping will be used to analyse uncertainty. Uncertainty inherent in the data will be represented by means of a cost-effectiveness acceptability curve (CEAC). Analyses will be performed using MS Excel, SPSS and STATA.

### Process evaluation analysis

Full details of the process evaluation analysis are reported in Additional file 1.

The relationship between the quantitative and qualitative data will be examined [43].

### Methods in analysis to handle protocol non-adherence and any statistical methods to handle missing data {20c}

We will use chi-squared tests to compare follow-up rates between the three study groups to establish whether there is differential drop out. We will then explore the effect of alternative assumptions about the pattern of missing data, through complete case analysis and through using imputation methods [44].

We will report withdrawal from the intervention and from the study and reasons for withdrawal, where known.

### Plans to give access to the full protocol, participant-level data and statistical code {31c}

This document is the full protocol. To access the participant-level dataset or statistical code, please contact the corresponding author (MU).

### Oversight and monitoring

#### Composition of the coordinating centre and trial steering committee {5d}

The Chief Investigator (CI) will have overall responsibility for the study and its management. The Trial Management Group (TMG) at the University of Stirling, including the CI, a statistician, data manager and researcher, will be responsible for the day-to-day running of the trial, as well as overseeing data management and analyses. The TMG will meet as needed and will be supported by and report to a Trial Steering Committee (TSC). The TSC will have an independent chairperson and members but also include the TMG, other trial investigators and a representative of the funders. The TSC will meet every 6 months and more often if required. In addition, the site Principal Investigators (PIs) will meet with the Chief Investigator (CI) every 3 months to discuss progress. The CI will send a monthly newsletter, including recruitment and follow-up rates, to the TMG, to all co-investigators and to all site staff.

#### Composition of the data monitoring committee, its role and reporting structure {21a}

A separate Data Monitoring and Ethics Committee is not judged necessary, as we cannot envisage the intervention having the potential to harm participants.

### Adverse event reporting and harms {22}

It is not considered necessary to record or report adverse events as the intervention being tested involves the offer of financial incentives (Lovetoshop vouchers) which could not be a contributory factor in adverse events.

### Frequency and plans for auditing trial conduct {23}

The Trial Steering Committee will meet at least every 6 months to audit trial conduct and progress. This will include independent monitoring of adherence to the study protocol; approving changes to the study protocol; reviewing quality assurance indicators; monitoring study recruitment and the overall timetable; advising, as required, on specific scientific items that may arise; compliance with legislation; adherence to research governance; reporting to funders; and approving publication and dissemination strategies.

### Plans for communicating important protocol amendments to relevant parties (e.g. trial participants, ethical committees) {25}

Amendments will be approved by the research ethics committee and Health Research Authority. Funders, sponsors and NHS Research and Development Offices will be routinely informed of any amendments.

### Dissemination plans {31a}

In addition to journal publications and conference presentations, we will develop a publication and dissemination policy and will discuss presentations and dissemination with relevant patient and clinical interest groups.

## Discussion

At present, the majority of women who stop smoking in pregnancy return to smoking within 12 months of the birth of their baby. In the UK, there is currently no standard offer of help to support relapse prevention and no interventions have been shown to be effective. Incentive payments to maintain abstinence from smoking may provide a substantial benefit by reducing harmful health consequences for the mother and child and thereby reducing long-term health care costs. The results of this definitive trial should provide sufficient data to determine whether it is effective to offer financial incentives to women to help them avoid relapse to smoking. This evidence will provide information required for NICE to consider recommending financial voucher incentive payments to support pregnant smokers across the UK to maintain abstinence from the smoking after the birth of their baby.

### Trial status

The FIPPS trial is currently recruiting in four UK centres/Hospital Trusts in Greater Manchester. Recruitment began in February 2019 and is due to end In August 2021, with follow-up completed by September 2022. The trial has a TSC which has convened five times. During COVID-19, due to a combination of lack face-to-face screening of women’s smoking status in pregnancy and reduced staffing due to illness or ‘shielding’, fewer women have joined the pregnancy cessation scheme than expected and therefore there has been a smaller pool of women to screen to join the trial. Thus, the trial is behind target and will not recruit the target of 900 women by August 2021. As of June 30th, 2021, 424 participants have been recruited. Based on recent recruitment rates, we anticipate recruiting approximately 462 women by the end of August 2021. We do not have the resources to continue recruiting past this date. This has implications for the power calculations. Assuming *N* = 154 in each of the three study groups and effect sizes as estimated in the original sample size calculation, then the power for the three comparisons, all with an alpha threshold of 0.017, will be:

60% power for 3-month incentives versus 12-month incentive group comparison

82% power for the no incentives versus 3-month incentive group comparison

100% power for the no incentives versus 12-month incentive group comparison

The power for examining the significance (*p* < 0.05) of a linear contrast across the three groups from control to 12-month intervention will be 100%.

This article is based on protocol version V7.0, 16th November 2020. For the registered trial protocol and updates see 10.1186/ISRCTN55218215

## Supplementary Information

**Additional file 1.** Details of the process evaluation.

## Abbreviations

BCT: Behaviour change technique
CEAC: Cost-effectiveness acceptability curve
CI: Chief Investigator
CO: Carbon monoxide
CONSORT: Consolidated Standards of Reporting Trials
CRF: Case report form
GCP: Good Clinical Practice
GDPR: General Data Protection Regulation
GP: General Physicians
iCO: Bedfont single-person, self-administered carbon monoxide monitor
ICERs: Cost-effectiveness ratios
NICE: National Institute for Health and Care Excellence
NHS: National Health Services
NRT: Nicotine replacement therapy
PI: Principal Investigator
PIS: Participant information sheet
ppm: Parts per million
RCT: Randomised controlled trial
SOS: Significant Other Supporter
SSS: Stop smoking service
TMG: Trial management group
TSC: Trial Steering Committee
