# Supplementary material for: Financial Incentives for Preventing Postpartum return to Smoking (FIPPS): study protocol for a three-arm randomised controlled trial
Source: Trials. 2021 Aug 2;22:512. doi: 10.1186/s13063-021-05480-6 (PMC8327045; doi:10.1186/s13063-021-05480-6)
Supplement: Supplementary file 1 — Additional file 1. Details of the process evaluation. [file 13063_2021_5480_MOESM1_ESM.docx]

**Process evaluation**

Following MRC process evaluation guidance [1] both qualitative and quantitative data will be collected to assess implementation of the intervention, mechanisms of impact and contextual influences. The specific objectives of the process evaluation are to examine: the views of participants on the intervention; the views of those delivering the intervention on the intervention; study how the intervention is implemented; distinguish between components of the intervention; investigate contextual factors across different sites that affect the intervention; and study the way effects vary in subgroups. Quantitative data will include recruitment and follow-up rates. A qualitative descriptive methodology will elicit in-depth account of views and experiences of both those delivering the intervention and those receiving the intervention.

**(i) Interviews with trial participants**

**Aim of interviews:** To identify barriers and facilitators to trial recruitment and adherence and to explore the acceptability of study processes and procedures.

Recruitment and consent

We aim to recruit ten women from each of the three trial arms, after they have completed the 12-month postpartum follow-up. At the time of recruitment into the trial, all women will be asked by the SSS advisor if they consent to be contacted by a researcher, after the 12-month follow-up, who will invite them to be interviewed about their experiences of and views on the trial and intervention. Women who consent to be contacted will be reminded of this if they attend the 12 months follow-up. A researcher will then telephone the woman to answer any questions about the study and to obtain verbal informed consent to be interviewed. The semi-structured interview, lasting up to 30 minutes, can take place at this time or at another more convenient time, with the aim of completing within one month of the 12-month postpartum follow-up.

**Sample size and purposive sampling**

We will aim for a total sample size of 30. Using the ‘ten plus three’ rule for data saturation, a target of ten participants was set for each of the three trials arms, to achieve a point where three consecutive interviews have been conducted without new themes emerging. As far as possible, within each study group, sampling for interviews will be based on maximum diversity, principally, in relation to quit status (i.e., whether they were abstinent at 3 months and 12 months, abstinent at 3 months but not at 12 months, or not abstinent at 3 months), and also in relation to hospital Trust, age, socio-economic background and ethnicity. We will invite consecutive women who agree to be interviewed until we have achieved the target sample and achieved data saturation.

**Theoretical framework, topic guides and interviews**

The interviews will be conducted via telephone. Although telephone interviews have been criticised for compromising rapport and missing non-verbal cues, they are also thought to allow participants the opportunity to openly disclose sensitive material in a more anonymous manner compared with face-to-face [2]. We have used telephone interviews successfully in previous studies with pregnant and postpartum women and as the interviews were conducted during the COVID-19 pandemic a telephone method presented the most practical option.

Interviews will be arranged and conducted by two female qualitative researchers (JM, LM, non-smokers). The interviews will explore study processes and experiences of participating, including reasons for taking part and dropping out, experience of recruitment and randomisation (e.g., expectations and understanding of the study and its aims) and views on the incentives intervention and smoking assessment.

While allowing participants to speak freely, we will use the Theoretical Domains Framework (TDF) to inform the interview topic guide and data analysis. The TDF defines 14 domains into which all determinants of health behaviour change can be organized (e.g., beliefs about consequences, social influences, goals) [3,4]. When developing the interview topic-guide/questions we will use the TDF to ensure broad coverage of the components that drive smoking cessation. Analysis will also be informed by the Capability-Opportunity-Motivation-Behaviour (COM-B) framework [5].

The topic guides will be reviewed by two Patient and Public Involvement representatives and by the wider study team. Interviews, will be digitally recorded and transcribed verbatim by professional transcribers. The resulting transcripts will be anonymised. To compensate the women for their time, and as a gesture of thanks, they will be offered £30 in Love2Shop vouchers.

The interviews will be digitally-recorded and transcribed verbatim by an independent transcriber. The analysis will be both deductive, through being informed by the TDF and the topics chosen for the interviews, and inductive, from the accounts of participants. The analysis will be undertaken in five stages, using a thematic approach [6]:

(i) Reading the transcripts several times to ensure familiarity with data and identify emerging themes.

(ii) Development of a coding frame work, which is informed by the TDF.

(iii) Verification of coding whereby two researchers independently code 10% of randomly selected transcripts from each smoking status group. If coding consistency is suboptimal, a further 10% of transcripts will be independently coded. All remaining transcripts will be coded once coding consistency is satisfactory.

(iv) Codes will be grouped into clusters that reflect broader themes.

(v) Discussion within the wider research team will be used to refine, label and interpret the themes and sub-themes.

The analysis will be assisted by QSR NVivo (v12) software and the results will be reported according to the consolidated criteria for reporting qualitative research tool [7].

**(ii) Focus group with stop Smoking Service Managers/Advisors delivering the intervention**

**Aims of focus group:** To identify barriers and facilitators to trial recruitment and adherence; to explore the acceptability of study processes and procedures; and to assess contextual influences at different recruiting sites, particularly those influences that might have affected the quality of the implementation of the intervention*.*

**Recruitment, consent and sampling**

When first joining the study, all SSS advisors who are delivering the trial interventions and managers overseeing the trial at each Trusts, will be given brief information about the focus group. At least 12 months after commencing recruitment, to ensure all the advisors and managers have sufficient experience of the trial, all advisors and managers will be sent a participant information sheet and will be invited to take part in a focus group, lasting up to 45 mins. Around 10 individuals will be selected to join the focus group and will give verbal consent. We will aim to include at last two individuals from each of the four Hospital Trusts, and invite advisors with the most experience of recruiting women to the trial and of delivering the interventions.

**Theoretical framework, topic guides and focus group**

The focus group will be arranged and conducted by the same two female qualitative researchers (JM, LM) who conducted the interviews with trial participants. The focus group will explore complementary topics to those presented in interviews with trial participants, including barriers and facilitators to conducting the trial (local capacity, organisational structures and any changes to these); and fidelity to trial processes (recruitment to the trial, information given to patients, training issues). The focus group topic guide and analysis will also be informed by both the TDF and the Normalization Process Model (NPM) [8,9,10]. The NPM is an evaluation model that asks what individuals and organisations do to make a complex intervention workable and to integrate it in routine practice. The NPM addresses how individuals understand and come to engage and support a new practice and how they reflect on and evaluate it. For example, this includes exploration of the following processes the SSS would have to go through in order to routinely embed the intervention: to fully understand the nature of the incentives intervention and how it works, to engage other stakeholders (e.g., maternity services) with the intervention, the work the SSS will have to undertake to enact the intervention, and the evaluation of the intervention and its impact. Within the umbrella of this model, focus group topics will include: the need for co-operative work practices, accountability, allocation and evaluation of work tasks, the link between work related to the incentives intervention and existing organisational structures, procedures and interventions and the need for allocating and organizing resources in order to integrate the intervention into routine SSS practice.

The focus group will be digitally-recorded and transcribed verbatim by an independent transcriber. The resulting transcripts will be anonymised. The analysis will be both deductive, through being informed by the TDF and NPM and the topics chosen for the focus group, and inductive from the accounts of participants. The analysis method will be the same as for the interviews with trial participants, except the coding framework will be informed by the NPM as well as by the TDF.

**References**

1. Moore GF, Audrey S, Barker M, Bond L, Bonell C, Hardeman W, Moore L, O'Cathain A, Tinati T, Wight D, Baird J. [Process evaluation of complex interventions: Medical Research Council guidance.](https://pubmed.ncbi.nlm.nih.gov/25791983/) BMJ. 2015;350:h1258.

2. Novick G. Is there a bias against telephone interviews in qualitative research? Res Nurs Health. 2008;31:391-8.

3. Cane J, O’Connor D, Michie S. Validation of the theoretical domains framework for use in behaviour change and implementation research. Implement Sci. 2012;7:37.

4. Michie S, Johnston M, Abraham C, Lawton R, Parker D, Walker A. Making psychological theory useful for implementing evidence-based practice: a consensus approach. Qual Saf Health Care. 2005;14:26-33.

5. Michie S, van Stralen MM, West R. The behaviour change wheel: A new method for characterising and designing behaviour change interventions. Implementation Science. 2011;6:42.

6. Braun V, Clarke V. Using thematic analysis in psychology. Qual Res Psych. 2006;3:77–101.

7. Tong A, Sainsbury P, Craig J. Consolidated criteria for reporting qualitative research (COREQ): A 32-item checklist for interviews and focus groups. Int. J. Qual. Health Care. 2007;19:349-57.

8. May C. A rational model for assessing and evaluating complex interventions in health care. BMC Health Serv Res. 2006;6:86.

9. May CR, Mair FS, Dowrick CF, Finch TL. [Process evaluation for complex interventions in primary care: understanding trials using the normalization process model.](https://www.ncbi.nlm.nih.gov/pubmed/17650326) BMC Fam Pract. 2007;24:42.

10. [McEvoy R](https://www.ncbi.nlm.nih.gov/pubmed/?term=McEvoy%20R%5BAuthor%5D&cauthor=true&cauthor_uid=24383661), [Ballini L](https://www.ncbi.nlm.nih.gov/pubmed/?term=Ballini%20L%5BAuthor%5D&cauthor=true&cauthor_uid=24383661), [Maltoni S](https://www.ncbi.nlm.nih.gov/pubmed/?term=Maltoni%20S%5BAuthor%5D&cauthor=true&cauthor_uid=24383661), [O'Donnell CA](https://www.ncbi.nlm.nih.gov/pubmed/?term=O%27Donnell%20CA%5BAuthor%5D&cauthor=true&cauthor_uid=24383661), [Mair FS](https://www.ncbi.nlm.nih.gov/pubmed/?term=Mair%20FS%5BAuthor%5D&cauthor=true&cauthor_uid=24383661), [Macfarlane A](https://www.ncbi.nlm.nih.gov/pubmed/?term=Macfarlane%20A%5BAuthor%5D&cauthor=true&cauthor_uid=24383661). A qualitative systematic review of studies using the normalization process theory to research implementation processes. [Implement Sci.](https://www.ncbi.nlm.nih.gov/pubmed/?term=McEvoy+R+Ballini+L) 2014;9:2.
